# Non-Esterified Fatty Acids Profiling in Rheumatoid Arthritis: Associations with Clinical Features and Th1 Response

**DOI:** 10.1371/journal.pone.0159573

**Published:** 2016-08-03

**Authors:** Javier Rodríguez-Carrio, Mercedes Alperi-López, Patricia López, Francisco Javier Ballina-García, Ana Suárez

**Affiliations:** 1 Area of Immunology, Department of Functional Biology, University of Oviedo, Oviedo, Asturias, Spain; 2 Department of Rheumatology, Hospital Universitario Central de Asturias, Oviedo, Asturias, Spain; SERGAS (Servizo Galego de Saude) and IDIS (Instituto de Investigación Sanitaria de Santiago), the NEIRID Lab, Research Laboratory 9, Santiago University Clinical Hospital. Santiago de Compostela, SPAIN

## Abstract

**Objectives:**

Since lipid compounds are known to modulate the function of CD4^+^ T-cells and macrophages, we hypothesize that altered levels of serum non-esterified fatty acids (NEFA) may underlie rheumatoid arthritis (RA) pathogenesis.

**Methods:**

Serum levels of NEFA (palmitic, stearic, palmitoleic, oleic, linoleic, γ-linoleic, arachidonic –AA–, linolenic, eicosapentaenoic –EPA– and docosahexaenoic –DHA–) were quantified by LC-MS/MS after methyl-*tert*-butylether (MTBE)-extraction in 124 RA patients and 56 healthy controls (HC). CD4^+^ phenotype was studied by flow cytometry. TNFα, IL-8, VEGF, GM-CSF, IFNγ, IL-17, CCL2, CXCL10, leptin and resistin serum levels were quantified by immunoassays. The effect of FA on IFNγ production by PBMC was evaluated in vitro.

**Results:**

Lower levels of palmitic (p<0.0001), palmitoleic (p = 0.002), oleic (p = 0.010), arachidonic (p = 0.027), EPA (p<0.0001) and DHA (p<0.0001) were found in RA patients, some NEFA being altered at onset. Cluster analysis identified a NEFA profile (hallmarked by increased stearic and decreased EPA and DHA) overrepresented in RA patients compared to HC (p = 0.002), being associated with clinical features (RF, shared epitope and erosions), increased IFNγ expression in CD4^+^ T-cells (p = 0.002) and a Th1-enriched serum milieu (IFNγ, CCL2 and CXCL10, all p<0.005). In vitro assays demonstrated that imbalanced FA could underlie IFNγ production by CD4^+^ T-cells. Finally, changes on NEFA levels were associated with clinical response upon TNFα-blockade.

**Conclusion:**

An altered NEFA profile can be found in RA patients associated with clinical characteristics of aggressive disease and enhanced Th1 response. These results support the relevance of lipidomic studies in RA and provide a rationale for new therapeutic targets.

## Introduction

Rheumatoid Arthritis (RA) is a rheumatic condition associated with joint erosion and destruction caused by an immune response against self-antigens. Autoimmune mechanisms orchestrating its pathogenesis have been conventionally considered to be regulated by genes and proteins. However, in the era of the “omics” sciences, metabolomics -and especially lipidomics- are changing this dogma by the identification of novel players [1,2]. Among them, lipids are emerging as potential pivotal candidates.

Largely considered as simple energy stores and structural components, lipid compounds are currently known to actively control a number of biological processes. The notion that lipid species can shape the immune response is currently becoming accepted [3–5], although the exact players of this crosstalk remain largely unclear. Interestingly, changes in lipid depots are related to changes in gene expression and function in monocytes and CD4^+^ T-cells through secreted compounds [6–8], the non-esterified fatty acids (NEFA) being the main responsible of these effects. Accordingly, NEFA are known to be involved in a number of relevant processes for the immune system, such as modulation of the production of cytokines and chemokines [9–11], expression of adhesion molecules [12,13] as well as the release of proinflammatory and anti-inflammatory lipid-derived compounds [14,15]. Therefore, it is tempting to speculate that altered levels of NEFA would underlie a disturbed, poorly controlled, immune response. However, to what extent these mechanisms are relevant for human immune-mediated diseases is unknown.

Although some metabolomic studies have been performed in RA [16–18], little evidence on NEFA has been reported. Additionally, no standardized protocols are available for NEFA analysis, and lipid analyses have been accompanied by technical issues only partly overcome recent years [19]. Actually, results on NEFA until date show a considerable variation even within healthy (control) populations. Recently, some authors have revealed the presence of different NEFA on synovial fluid (SF) in RA patients, but the clinical relevance of these findings was not studied [20]. Consequently, which the serum NEFA profile in RA patients is and whether it could be associated with clinical features remain unknown. Elucidation of serum NEFA profile in RA would provide new insight into the pathological mechanisms underlying this condition and it would also lead to the identification of novel biomarkers for clinical management. Therefore, the main aims of the present study were (i) to analyze the levels of serum NEFA in RA patients, (ii) to investigate their association with clinical features and (iii) to elucidate whether NEFA profiles may be linked to the impaired CD4^+^ T-cell response in RA.

## Methods

### Ethics approval

Approval for the study was obtained from the Institutional Review Board (Comité Regional de Investigación Clínica del Principado de Asturias), in compliance with the Declaration of Helsinki. All the participants gave written informed consent prior to their inclusion in the study. All methods were carried out in accordance with the approved guidelines.

### Patients and controls

Our study involved 124 RA patients recruited from the Department of Rheumatology at Hospital Universitario Central de Asturias. All of them fulfilled the 2010 American College of Rheumatology classification criteria for RA. Some RA patients were recruited when the clinical diagnosis of RA was established, thereby exhibiting no previous exposure to any treatment (RA onset, n = 18). A complete clinical examination, including Disease Activity Score 28-joints (DAS28) calculation, was performed on each patient during their clinic appointment and an overnight fasting blood sample was drawn by venipuncture. Additionally, 13 biologic-naïve RA patients (12 women, median age 43 (range: 30–65), DAS28 5.08(1.93), 38.5% RF+, 46.1% ACPA+), candidates for anti-TNFα therapy, were prospectively followed for three months. Blood samples were collected immediately before and 3 months after TNFα-blocking therapy. Clinical response was evaluated by EULAR criteria.

Simultaneously, 56 age- and gender- and BMI-matched healthy individuals (HC) were recruited from the general population (42 female, age (mean±SD): 48.98±7.75 years, BMI: 25.8(4.58)). Automated serum lipids analysis was carried out on all the participants from fasting fresh blood samples. Serum samples were stored at -80°C until analyses.

### NEFA extraction

A new methyl-*tert*-butylether (MTBE)-based extraction protocol was developed following Pizarro et al. [19,21]. Briefly, to 50 μl serum 5 μl of internal standard (300 ppm heptadecanoic acid) and 150 μl methanol chromasolv grade (Sigma) were added and tubes were vortexed for 30 s. Then, 750 μl MTBE chromasolv grade (Sigma) were added, vortexed and an incubation for 30 minutes in a ultrasound (US)-assisted water bath at 15°C was performed. For phase separation, 125 μl milliQ water were added, vortexed and centrifuged for 7 minutes at 5000 rpm (15°C). Organic phase was collected and the extraction steps were repeated once with 100 μl MetOH, 300 μl MTBE and 100 μl milliQ H_2_O.

Lipid extracts were dried in a miVac centrifugal evaporator (Genevac Ltd), and redissolved in 500 μl of water:acetonitrile 38:62.

### LC-MS/MS analysis

For the determination of the fatty acids, an Agilent 1290 Infinity HPLC system (Santa Clara), consisting of a high pressure binary pump, an autosampler and a column oven, was used. The column was a Zorbax Eclipse Plus (Agilent) C18, 50x2.1 mm, 1.8 μm. Mobile phases A and B were water and acetonitrile respectively, both containing formic acid 0.1%. Fatty acids separation was carried out by the following gradient program: 62% B (held for 4.5 min) followed by a linear increase up to 100% B in 10 min (held for 1 min). The column temperature was set at 45°C and the injection volume was 2 μl.

Mass detection was performed using an Agilent 6460 triple quadrupole mass spectrometer with electrospray ionization, operated in the negative SIM mode (S1 Fig). The settings of the mass spectrometer were as follows: spray voltage 3.5 kV; gas flow 9 l/min; gas temperature 300° C; nebulizer pressure 50 psi; sheath gas flow 11 l/min at 350° C.

For quantitation, calibration curves for each compound were prepared by proper dissolution of the pure (>99%) standards (all from Sigma) in methanol to encompass the expected concentration of the analytes in the sample. The calibration ranges were 0.4–12.5 μg/ml for EPA and γ-linolenic; 1.2–37.5 μg/ml for DHA and linolenic; 2.3–75 μg/ml for AA and palmitoleic; 7.8–250 μg/ml for oleic and linoleic and 15.6–500 μg/ml for palmitic and stearic. Detection limits were as follows: 0.10 μg/ml EPA, 0.46 μg/ml DHA, 0.01 μg/ml AA, 0.01 μg/ml γ-linolenic, 0.42 μg/ml linolenic, 0.04 μg/ml palmitoleic, 0.45 μg/ml linoleic, 0.34 μg/ml palmitic, 1.01 μg/ml oleic and 1.91 μg/ml stearic. A good linearity was observed in all cases (r^2^>0.994). Internal standard was used to correct for potential variations during the sample preparation step.

### Total NEFA measurement

Total serum NEFA level was measured using an enzymatic colorimetric assay (NEFA kit, Roche). Maxisorp Nunc plates were used and absorbance was recorded at 546 nm, following manufacturer instructions. Standard curves between 1.50 and 0.02 mM were included and intra- and inter-assay coefficients of variation were 9.42 and 10.0%, respectively.

### Flow cytometry analysis

CD4^+^ phenotype was analyzed by flow cytometry as previously described [22]. Briefly, 300 μl peripheral blood were stained with anti-CD4 APC-Cy7 (Immunostep) and anti-CD25 FITC for 30 minutes at 4°C. Then, erythrocytes were lysed and cells were fixed and permeabilized (FOXP3 transcription factor staining kit, eBioscience) and intracellularly stained with anti-FOXP3 PE, anti-IFNγ PerCP-Cy 5.5 and anti-IL17 APC (all eBiosciences) or isotype controls (eBioscience). Samples were analyzed and 50,000 CD4^+^ lymphocytes were acquired in a FACS Canto II (BD) cytometer. CD4^+^ cells were gated and their intensity of IFNγ and IL-17 intracellular staining (measured as mean fluorescence intensity, MFI) was analyzed with FACS Diva v. 2.6 (BD) by subtracting the MFI in the isotype control tube to that of the specific antibody-stained one. Regulatory T cells (Treg) were identified as CD4^+^CD25^high^FOXP3^+^.

### Culture assays

Peripheral blood mononuclear cells (PBMC) were isolated by centrifugation on Ficoll (Lymphoprep, PAA) gradients from buffy coat preparations obtained from healthy donors and washed twice with sterile PBS. Then, cells were cultured for 48 h in 48-well plates at 2·10^6^ cells/ml in RPMI 1640 (Bio Witthaker) with 10% heat-inactivated FCS (PAA) and 100 μg/ml streptomycin and ampicillin (Sigma) in the presence of different concentrations of stearic, DHA and EPA fatty acids dissolved in DMSO (Sigma) under resting conditions or with 2.5 μg/ml phytohaemagglutinin A (PHA) (Sigma). Negative controls were treated with the same amount of DMSO. FA concentrations (stearic and DHA: 25, 50 and 100 μM; EPA: 25 and 50 μM) were determined by previous experiments among those not exhibiting cytotoxic effects. Parallel cultures were performed either to analyze culture supernatants or intracellular production of cytokines. To assess the production of cytokines, cells were treated with 20 ng/ml phorbol-12-myristate-13-acetate (PMA), 500 ng/ml ionomycin and 2 μM monensin (all from Sigma) for 5 hours. Then, cells were processed as previously described for flow cytometry analysis of intracellular IFNγ accumulation. Propidium iodide was used to exclude non-viable cells.

### Quantification of cytokines

IFNγ levels were analyzed in serum samples and culture supernatants with an OptEIA kit (BD) following manufacturer’s instructions. Detection limit was 0.58 pg/ml. Serum levels of IL-8, VEGF, IL-17 and GM-CSF were measured with a CBA kit (BD) according to developer’s recommendations. Detection limits were 1.2 pg/ml, 4.5 pg/ml, 0.3pg/ml and 0.2 pg/ml, respectively. TNFα, CCL2 (MCP-1), CXCL10 (IP-10), Resistin and Leptin were assessed with Mini-EDK ELISA kits (Peprotech). Detection limits were 3.9 pg/ml, 8 pg/ml, 3.9 pg/ml, 63 pg/ml and 24 pg/ml, respectively.

### Statistical analysis

Continuous variables are summarized as median (interquartile range) or mean ± standard deviation whereas n(%) was used for categorical ones. Differences between two groups were evaluated by Mann Withney U tests, whereas Kruskal-Wallis or one-way ANOVA were chosen when more than two groups were included. Dunn-Bonferroni or Bonferroni corrections for multiple comparisons were carried out when Kruskal-Wallis tests exhibited significant differences, and p-values for each comparison are shown. Categorical variables were analyzed by χ^2^ square or Fisher exact tests. Paired t tests were performed on prospective samples. Correlations were analyzed by Spearman’s rank test. Principal Component Analysis (PCA) with Varimax rotation was performed. The number of components retained was based on eightenvalues (>1) and loadings greater than 0.5 were used to identify the variables comprising a single component. For cluster analysis, squared euclidean distances were computed from PCA scores in order to avoid biases due to redundant information and differences in ranges among variables, and Ward’s Minimum Variance Method was used to identify clusters minimizing the loss of information. Heatmap were built with R package *heatmap*.*2* for visualization purposes. Findings from correlation analyses and size effect (Hedges’s statistic) were considered to choose candidate individual FA for in vitro assays. SPSS 19.0, R 3.0.3 and GraphPad Prism 5.0 for Windows were used.

## Results

### NEFA levels in HC and RA patients

Individual and total serum NEFA levels were measured in 124 RA patients (Table 1) and 56 healthy controls (HC) and the results are summarized in Table 2. Total NEFA levels were similar in patients and controls but palmitic, palmitoleic, oleic, AA, EPA and DHA were decreased in RA patients. Differences were unrelated to age, gender or BMI. No effect of disease activity on NEFA levels was detected in RA, but disease duration was positively correlated with stearic levels (r = 0.364, p<0.001) and negatively with palmitic (r = -0.252, p = 0.007), palmitoleic (r = -0.236, p = 0.012), AA (r = -0.389, p<0.001), EPA (r = -0.243, p = 0.009) and DHA (r = -0.260, p = 0.006) (S2 Fig). However, linoleic and DHA were impaired in patients without previous exposure to treatments (RA onset) (S1 Fig), thereby supporting that other factors than disease duration are responsible for the NEFA disturbances in RA. Interestingly, higher levels of stearic were found in patients with established disease compared to their early counterparts (p = 0.016).

**Table 1:** Demographic and clinical parameters of RA patients.

	RA patients(n = 124)
Gender (female:male)	99:25
Age at sampling, years (mean±SD)	52.47 ± 12.76
*Disease features*	
Disease duration, years, median (range)	4.91 (0.00–30.00)
Age at diagnosis, years (mean±SD)	46.39 ± 12.70
Recruited at onset, n(%)	18 (14.5)
BMI	26.43 (6.38)
Disease activity (DAS28)	3.70 (2.08)
Tender Joint Count	2.00 (7.50)
Swollen Joint Count	1.50 (5.00)
Patient Global Assessment (0–100)	42.00 (40.00)
ESR, mm/h	16.00 (22.75)
CRP, mg/l	2.00 (4.00)
HAQ (0–3)	0.93 (1.22)
RF (+), n(%)	76 (61.2)
ACPA (+), n(%)	74 (59.6)
ANA (+), n(%)	68 (54.8)
Shared epitope, n(%) [n = 91]	54 (59.3)
Shared epitope (2 copies), n(%) [n = 91]	13 (14.2)
Erosive disease, n(%)	48 (38.7)
*Traditional CV risk factors*, *n(%)*	
Dyslipidemia	43 (34.6)
Hypertension	40 (32.2)
Diabetes	11 (8.8)
Obesity (BMI>30)	26 (20.9)
Smoking habit	43 (34.6)
Previous CV events	18 (14.5)
*Treatments*, *n(%)*^*‡*^	
Glucocorticoids	56 (52.8)
Methotrexate	87 (82.0)
TNFα blockers	44 (41.5)
Tocilizumab	12 (11.3)
Statins	23 (21.6)

Continuous variables are summarized as median (interquartile range) and n(%) was used for categorical ones, unless otherwise was stated.

^*‡*^ Frequency of treatments are calculated excluding patients recruited at diagnosis (untreated, n = 18) (n = 106)

**Table 2:** Individual and total NEFA serum levels in the study participants.

NEFA (μg/ml)	HC(n = 56)	RA patients(n = 124)	*p-value*
Palmitic (16:0)	1624.48 (763.33)	1000.69 (714.19)	< 0.0001
Stearic (18:0)	311.70 (118.84)	317.65 (79.47)	0.149
Palmitoleic (16:1w7)	14.67 (9.56)	10.09 (7.50)	0.002
Oleic (18:1w9)	297.79 (153.51)	209.21 (230.51)	0.010
Linoleic (18:2w6)	200.41 (143.00)	153.79 (156.60)	0.084
γ-linoleic (18:3w6)	1.44 (0.27)	1.42 (0.45)	0.920
AA (20:4w6)	11.16 (4.93)	9.27 (5.11)	0.027
Linolenic (18:3w3)	9.29 (3.74)	7.95 (5.05)	0.069
EPA (20:5w3)	2.62 (1.11)	1.90 (1.03)	< 0.0001
DHA (22:6w3)	12.55 (11.69)	7.80 (6.26)	< 0.0001
Total NEFA (mM)	0.41 (0.19)	0.47 (0.34)	0.157

Serum levels of individual NEFA (μg/ml, measured by LC-MS/MS) and total NEFA (mM, measured by an enzymatic colorimetric assay) are summarized as median (interquartile range) and differences were assessed by Mann Withney U test.

Differences in the individual NEFA remained after correction by the total NEFA level, thus pointing to an altered NEFA profile in RA patients. Of note, altered FA exhibited diverse chain length and double bound location, so impairment of NEFA levels cannot be solely attributed to their chemical properties.

Since a number of correlations between individual NEFA levels were found, we conducted a PCA on the NEFA in order to exclude potential collinearity biases and avoid loss of information. All NEFA showed communalities higher than 0.5, and both the Kaiser-Meyer-Olkin test (0.716) and the Bartlett test of sphericity (p = 10^−123^) provided a good adequacy of the data and explained 77.58% of the total variation. PCA resulted in 4 components: C1 (including AA, EPA and DHA), C2 (including palmitic, oleic, linoleic and γ-linolenic), C3 (including palmitoleic and linolenic) and C4 including (stearic). Therefore, PCA analysis revealed that individual NEFA within a given class seem to follow distinct patterns and similar patterns were found among different groups of NEFA.

### NEFA profile in RA patients and its associations with clinical features

Since our results pointed to an altered NEFA profile in RA, we wondered whether it could be found in the whole RA population or if, on the contrary, NEFA profiles may be associated with specific clinical characteristics. To this aim, a cluster analysis was performed from PCA scores. Results from the cluster analyses revealed the existence of two groups with different pattern and levels of individual NEFA, so we referred to them as NEFA^high^ and NEFA^low^ profiles.

**Fig 1:**
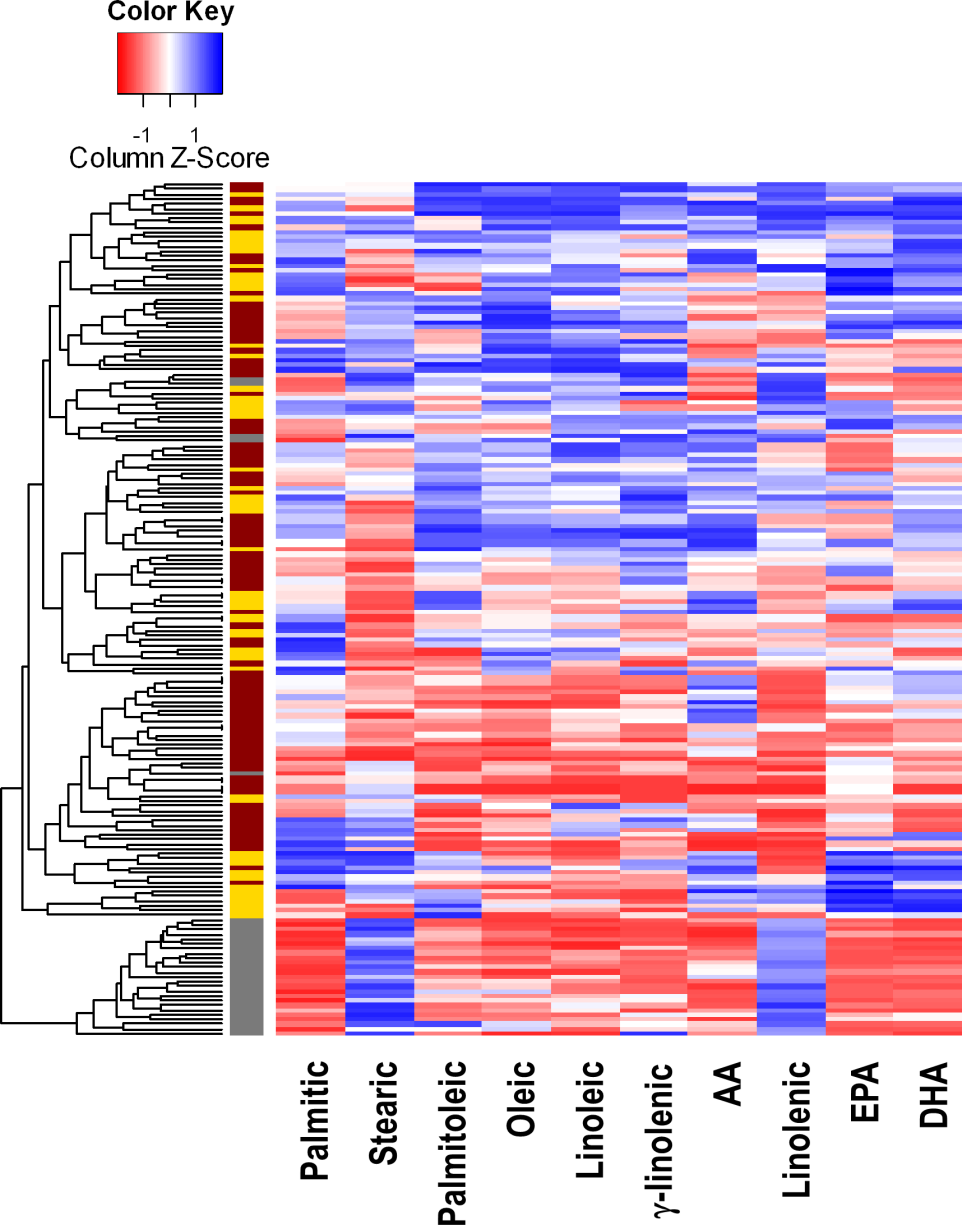
NEFA levels in HC and RA patients. Heatmap of NEFA serum levels showing the different NEFA species (columns). Each row corresponds with an individual. Colours in the vertical bar at the left of the heatmap identifies healthy controls (yellow), NEFA^high^ RA patients (brown) and NEFA^low^ RA patients (gray). Since NEFA^low^ profile was very infrequent in HC, controls were not divided into NEFA profiles. Tiles are coloured based on serum NEFA concentrations, red and blue indicating low or high levels, respectively.

Interestingly, the distribution of NEFA profiles was different between patients and HC, the NEFA^low^ profile being almost absent in HC (3/56) compared to the RA group (30/124, p = 0.002). When NEFA levels were plotted in a heatmap (Fig 1), HC and NEFA^high^ RA patients clustered together, whereas NEFA^low^ RA patients formed a separate group. NEFA^low^ profile was hallmarked by increased stearic levels in combination with decreased palmitic, EPA and DHA (S2 Table) (Fig 1). No differences in total NEFA levels were found among profiles in RA.

Next, we analyzed whether the NEFA profiles were associated with clinical features in RA patients. Whereas no differences in age, gender, traditional CV risk factors or treatments were observed, the NEFA^low^ profile was associated with higher disease duration, RF positivity and erosive disease (Table 3). Moreover, patients carrying two copies of the shared epitope were more likely to exhibit a NEFA^low^ profile. Since these associations may be attributed to the longer disease duration, patients at onset (n = 18) were excluded from the analysis, but the associations remain significant. Overall, these results suggest that a specific NEFA profile is found in RA linked to markers of aggressive disease.

**Table 3:** NEFA profiles and clinical features.

	NEFA^high^(n = 94)	NEFA^low^(n = 30)	*p-value*
Gender (female:male)	75:19	24:6	0.980
Age at sampling, years (mean±SD)	52.29 ± 13.65	52.86 ± 11.34	0.949
*Disease features*			
Disease duration, years, median (range)	3.83 (0.00–30.00)	7.04 (2.17–17.50)	0.002
Age at diagnosis, years (mean±SD)	46.92 ± 13.46	44.90 ± 10.32	0.325
Recruited at onset, n(%)	18 (19.1)	0 (0.0)	
BMI	26.43 (6.83)	26.37 (6.57)	0.310
Disease activity (DAS28)	3.88 (2.40)	3.52 (1.36)	0.230
Tender Joint Count	3.00 (7.00)	1.50 (5.00)	0.064
Swollen Joint Count	2.00 (5.00)	0.00 (2.50)	0.059
Patient Global Assessment (0–100)	47.00 (45.00)	37.50 (38.50)	0.087
ESR, mm/h	18.00 (23.50)	12.00 (22.00)	0.600
CRP, mg/l	1.65 (4.00)	2.30 (4.28)	0.244
HAQ (0–3)	1.00 (1.16)	0.87 (1.15)	0.173
RF (+), n(%)	52 (55.3)	24 (80.0)	0.029
ACPA (+), n(%)	52 (55.3)	22 (73.3)	0.184
ANA (+), n(%)	48 (51.0)	20 (66.6)	0.202
Shared epitope, n(%) [n = 91]	43 (60.5)	11 (55.0)	0.655
Shared epitope (2 copies), n(%) [n = 91]	5 (7.0)	8 (40.0)	<0.001
Erosive disease, n(%)	29 (30.8)	19 (63.3)	0.003
*Traditional CV risk factors*, *n(%)*			
Dyslipidemia	30 (31.9)	13 (43.3)	0.286
Hypertension	31 (32.9)	9 (30.0)	0.735
Diabetes	8 (8.5)	3 (10.0)	0.816
Obesity (BMI>30)	23 (24.4)	3 (10.0)	0.069
Smoking habit	29 (30.8)	14 (46.6)	0.122
Previous CV events	13 (13.8)	5 (16.6)	0.701
*Treatments*, *n(%)*^*‡*^			
Glucocorticoids	48 (63.1)	20 (66.6)	0.494
Methotrexate	63 (82.8)	24 (80.0)	0.521
TNFα blockers	28 (36.8)	16 (53.3)	0.163
Tocilizumab	7 (9.2)	5 (16.6)	0.297
Statins	16 (21.0)	7 (23.3)	0.634

Continuous variables are summarized as median (interquartile range) and n(%) was used for categorical ones, unless otherwise was stated. Differences were analyzed by Mann Withney U, χ^2^ square or Fisher exact tests, as appropriate.

^*‡*^ Frequency of treatments are calculated excluding patients recruited at diagnosis (untreated, n = 18) (NEFA^high^: n = 76, NEFA^low^: n = 30)

### NEFA profile in RA patients and immune parameters

Given the proposed role of certain FA as mediators of inflammatory responses, we explored whether increased Th1 or Th17 responses, usually found in RA patients, may be associated with NEFA profiles. Therefore, both effector (IFNγ^+^ or IL-17^+^) and regulatory (Foxp3^+^) CD4^+^ T-cells as well as cytokine serum levels were analyzed in patients and controls (Table 4).

**Table 4:** NEFA profiles and immune features.

	HC(n = 56)	NEFA^high^(n = 94)	NEFA^low^(n = 30)	*p-value(NEFA low vs high)*
*CD4*^*+*^ *phenotype*				
CD4^+^ T cells (%)	43.20 (14.76)	40.86 (17.86)	38.83 (12.54)	
MFI IFNγ (CD4^+^)	33.50 (31.00)	40.50 (33.00)	57.00 (19.00)***	0.002
MFI IL17 (CD4^+^)	44.85 (36.00)	45.00 (48.00)	54.00 (44.00)	
CD4^+^CD25^high^FOXP3^+^ Treg cells (%)	0.74 (0.47)	1.03 (0.65)	1.05 (0.71)	
*Serum cytokines*				
TNFα, pg/ml	93.94 (181.89)	240.21 (264.24)***	331.36 (346.39)***	0.946
IL-8, pg/ml	14.84 (14.83)	43.13 (16.46)***	43.48 (22.90)***	0.931
IL-17, pg/ml	1.07 (6.95)	1.14 (21.85)	2.25 (80.34)	
VEGF, pg/ml	103.89 (51.33)	113.34 (49.83)	118.08 (38.10)	
GM-CSF, pg/ml	21.33 (1.40)	26.24 (7.15)***	31.63 (15.56)***	< 0.001
IFNγ, pg/ml	3.29 (5.47)	3.66 (4.09)	5.90 (7.90)***	< 0.001
CCL2, pg/ml	244.50 (293.16)	252.97 (265.02)	931.49 (1089.94)***	< 0.0001
CXCL10, pg/ml	54.45 (41.40)	73.89 (104.80)*	147.75 (156.63)***	0.006
Leptin, ng/ml	7.57 (8.33)	12.35 (14.41)**	10.17 (10.22)*	0.371
Resistin, pg/ml	6.78 (3.38)	9.74 (5.03)***	9.83 (6.30)*	0.804

Variables are summarized as median (interquartile range) and differences were analyzed by Kruskal-Wallis (K-W) test with Dunn-Bonferroni correction for multiple comparisons tests. When Kruskal-Wallis test revealed differences among groups, p-values of post hoc tests between NEFA^low^ and NEFA^high^ profiles were indicated in the right column, whereas differences between each NEFA profile and HC are indicated as *p<0.050, **p<0.010, ***p<0.001.

Concerning the CD4^+^ phenotype, flow cytometry analyses showed that NEFA^low^ profile was related to increased IFNγ expression, but no associations were observed in the frequency of total CD4^+^ T-cells, Treg or IL-17 expression levels.

Although RA patients exhibited raised levels of proinflammatory cytokines compared to HC, some striking differences were noted between NEFA profiles. NEFA^low^ RA patients exhibited increased IFNγ serum levels, thus highlighting a link between NEFA profile and increased IFNγ production both at cellular and systemic level. NEFA^low^ profile was also related to increased CCL2 and CXCL10 (both Th1-related chemokines) as well as GM-CSF. Interestingly, IFNγ concentration paralleled levels of stearic acid (r = 0.416, p<0.001) whereas a negative association was found with EPA (r = -0.247, p = 0.009) and DHA (r = -0.263, p = 0.006) (S2 Fig). Therefore, all these results support an association between an increased Th1-related cytokines response in RA patients and their NEFA profile, stearic, DHA and EPA having a potential role.

### NEFA can modulate IFNγ production by CD4^+^ T-cells

To elucidate whether individual NEFA or an altered NEFA profile could promote an excessive IFNγ response, in vitro studies were conducted to evaluate the effect of individual NEFA which hallmarked the NEFA^low^ profile on the production of IFNγ by PBMC. Candidate NEFA were chosen within those which were more differentially present between groups (based on p-values and Hedges’g statistic as a measure of the size effect) and which were also associated with IFNγ in univariate analysis. Therefore, the effect of stearic, DHA and EPA was evaluated by analyzing IFNγ release and IFNγ-producing CD4^+^ T-cells in PBMC cultures.

**Fig 2:**
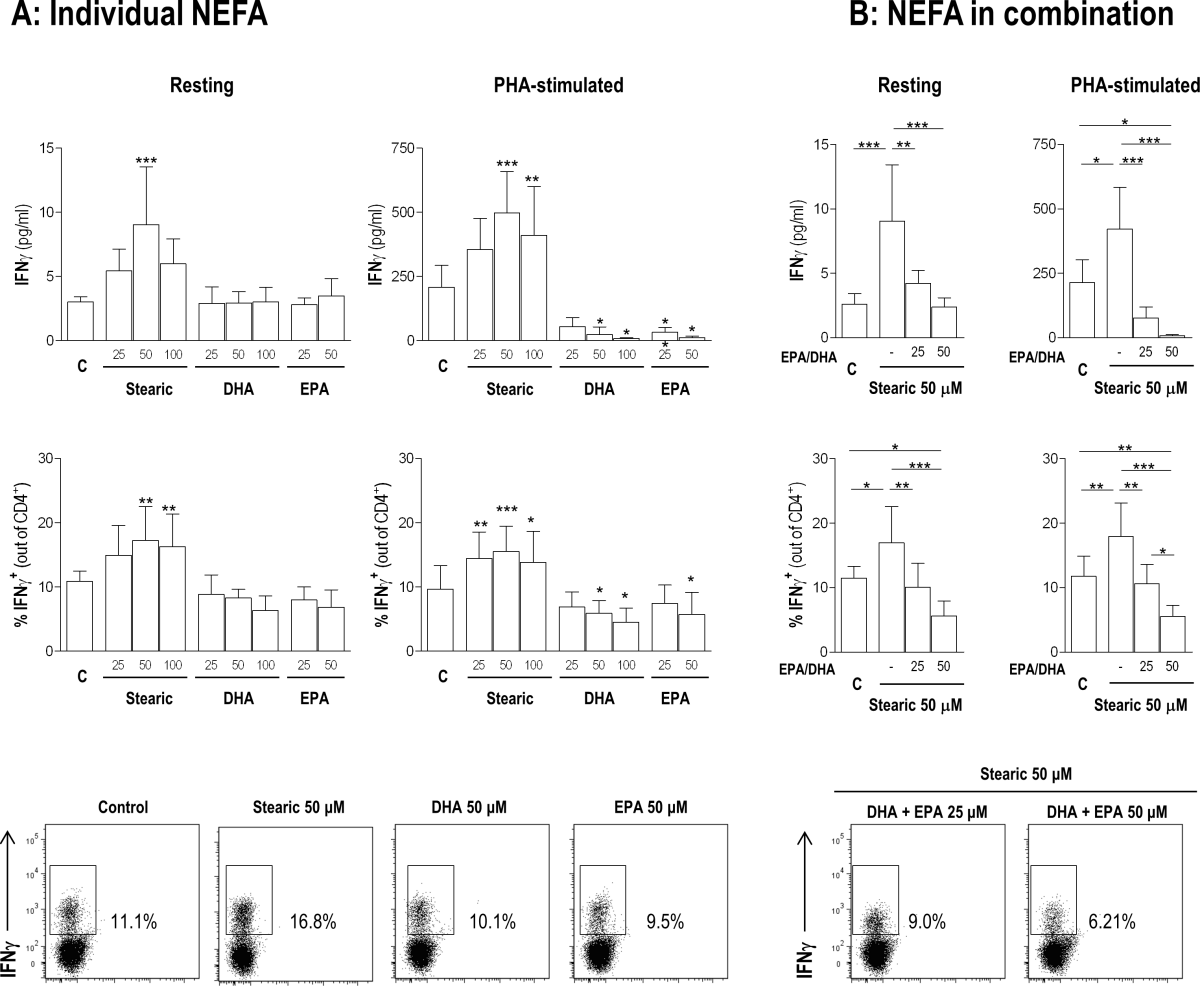
Effect of FA added alone or in combination on IFNγ production in vitro. **(A) Effect of the addition of individual FA to PBMC cultures.** The effect of individual FA (stearic, DHA and EPA) on IFNγ production by PBMCs under resting conditions or in the presence of PHA was evaluated in culture supernatants by ELISA or by flow cytometry on CD4^+^ T cells (both n = 6) **(B) Effect of FA added in combination to PBMC cultures.** The effect of different combinations of FA on IFNγ production was examined (n = 6). DHA and EPA were added at a final concentration of 25 or 50 μM each. Differences were assessed by ANOVA with Bonferroni multiple’s comparisons test and differences observed within pairs of groups are indicated as *p<0.050, **p<0.010 and ***p<0.001. Representative dot plots of flow cytometry quantification of IFNγ intracellular accumulation on gated CD4^+^ cells are shown. C: FA-untreated cells used as controls for each experiment (resting cells or PHA-stimulated cells).

After 48h of culture, stearic acid promoted an increase in IFNγ secretion in both resting and PHA-stimulated cells (Fig 2A). No effect for EPA and DHA was seen under resting conditions, although both NEFA inhibited PHA-stimulated IFNγ release. These findings were also noted when IFNγ accumulation in CD4^+^ T-cells was evaluated after PMA and ionomycin treatment of both resting and PHA-stimulated cells. No effect on CD4^+^ frequency or viability was observed at the concentrations tested (resting: p = 0.392 and p = 0.334; PHA: p = 0.998 and p = 0.292, respectively).

Therefore, stearic and EPA/DHA exhibited an opposite effect on IFNγ production that seems to be mediated, at least in part, by CD4^+^ T-cells. In order to evaluate the combined effect of these NEFA, PBMC were cultured in the presence of stearic acid (50 μM) alone or in combination with increasing amounts of both DHA and EPA (25 or 50 μM each). Our results clearly demonstrate that DHA and EPA were able to counteract the stearic-mediated IFNγ production in both resting and PHA-stimulated PBMC (Fig 2B). Again, this effect was observed at CD4^+^ T-cells level. Hence, our results support that the balance among stearic, DHA and EPA could influence the IFNγ-CD4^+^ response.

### NEFA profile upon therapeutic TNFα-blockade in RA patients

Finally, the changes on NEFA serum levels upon TNFα-blockade and its association with clinical outcome were evaluated. To this end, NEFA serum levels were analyzed in a subgroup of biological-naïve RA patients (n = 13) before and 3 months after TNFα-blockade initiation.

Anti-TNFα treatment was associated with decreasing EPA, DHA and AA levels in the whole group (S3 Table). However, when patients were stratified by EULAR clinical response, these NEFA declined in non-responders (NR, n = 8) but not in patients with a good clinical response (R, n = 5). On the contrary, stearic levels showed a decrease in patients who exhibited a good response but not in their NR-counterparts patients (S4 Table). Importantly, no differences in total NEFA levels were found.

Finally, although no effect of TNFα-blockade was observed on IFNγ serum levels (whole group: p = 0.152, NR: p = 0.401, R: p = 0.138), decreasing IFNγ expression on CD4^+^ T-cells was found in responders (91.00(33.00) vs 32.00(51.50), p = 0.043), but not in non-responders (p = 0.889). Interestingly, no differences in the total CD4^+^ frequency (43.14(13.84) vs 44.70(14.63)%, p = 0.074) or Treg subsets (0.94(0.60) vs 1.19(0.55)%, p = 0.170) were noted upon treatment.

These results suggest than decreasing EPA, DHA and AA levels are associated with poor response to TNFα inhibitors, whereas decreasing stearic levels did with a good response. Therefore, changes within NEFA serum pool may be connected with clinical outcome and IFNγ-CD4^+^ immune response in RA patients upon TNFα-blockade.

## Discussion

Several studies have recently focused on lipidomic approaches in a number on human diseases. Here we report the first results on NEFA quantification in RA and their associations with clinical features. Although other authors have previously performed a lipid profiling on SF from RA patients [20], our results went further by assessing the differences compared to a control population and their associations with clinical parameters, thus gaining insight on the clinical relevance of lipidomic profiling. Similarly, we focused on serum samples, which are easier to obtain and are more interesting to biomarker discovery than SF. Moreover, since systemic autoimmunity precede the synovial destruction [23], serum profiling exhibit additional advantages to unravel disease mechanisms. Finally, we have adapted an innovative method for NEFA extraction and analysis by LC-MS/MS from serum samples, with important advantages on efficiency, reproducibility, reliability and minimizing time-consuming steps [21]. Additionally, we have included an additional step to correct for total NEFA levels in serum by an independent technique, thus excluding the possibility that our results are based on differences in the total NEFA pool among individuals.

An important conclusion from our findings is the lack of a general pattern in NEFA impairment in RA, not being specific NEFA classes globally altered, but individual NEFA seemed to follow different patterns. The idea of different figures among same-class lipids was highlighted by other authors studying healthy adults [24], and put into question therapies based on lipid classes instead of individual lipid compounds.

The most outstanding result of our work is the identification of a NEFA profile associated with features of aggressive disease and increased Th1-related cytokines in RA patients. Similarly, altered expression of genes involved in FA metabolism has been reported in RA [25], as well as in individuals with pre-clinical RA [26]. Moreover, decreased ω3-FA content in erythrocytes was reported in HLA-DR4+ individuals with high risk of developing RA [27]. Accordingly, our results revealed altered NEFA levels, mainly EPA and DHA, in patients recruited an onset (medications-free) and also in association with SE, therefore suggesting that NEFA impairment could be an early event in RA, and not just a consequence of the disease course itself or the exposure to different treatments, thereby supporting the role of NEFA species in RA pathogenesis. Importantly, no effect of treatments was observed in the cross-sectional analysis. Although an association with glucocorticoid usage may have been expected, low doses of corticoids prescribed to RA patients may explain our findings.

Interestingly, the altered NEFA profile was linked to clinical characteristics but not to traditional CV risk factors, thereby excluding an effect of important metabolic alterations (such as diabetes or dyslipidemia) on the NEFA profiles found in RA. Then, particular alterations, rather than a global impairment on FA metabolism, may cause these profiles.

The NEFA^low^ profile seem to underlie changes in immune parameters, since increased IFNγ serum levels and IFNγ expression on CD4^+^ T-cells were found in NEFA^low^ patients. The fact that CCL2 and CXCL10 were also increased lead us to think that altered NEFA could underlie an excessive Th1 response. Although it is known that adipose tissue-infiltrating leukocytes exhibit a Th1 phenotype [28,29], and that adipocytes can modulate CD4^+^ T-cell function by secreted lipids in vitro [6], the clinical relevance of these findings is unknown. Our results are the first evidence that this effect can be elicited by specific NEFA in the systemic compartment in patients. However, the actual mechanisms by which NEFA drive these changes in CD4^+^ T-cells are not totally understood. Based on the current literature, it is tempting to speculate that peroxisome proliferator-activated receptors (PPAR) have a pivotal role. PPAR can bind poly-unsaturated FA and eicosanoids [30,31], and their activation lead to the inhibition of several inflammatory genes, including NFκB pathway [32–34]. Actually, PPAR-deficient mice exhibit an uncontrolled response to inflammatory stimuli [30]. Thus, decreased FA-mediated PPAR activation may account for the increased inflammatory response seen in NEFA^low^. Interestingly, IFNγ can be negatively regulated by PPAR [35] and NFκB [36] pathways. Conversely, in spite of the role of the Th17 responses in RA, no associations were detected with the NEFA profiles, either at the serum or the CD4+ T-cell level. Furthermore, whether the IFNγ production could be underlie by a Th1 skewed response or it is solely attributed to a Th1 upregulation, remains to be elucidated since the Th2 subset was not analyzed in the present study.

Therefore, lower EPA and DHA at RA onset, or even at preclinical stages, could be unable to counteract Th1 responses, especially in patients with particular clinical or genetic characteristics, leading to an exacerbated inflammation. Moreover, changes in NEFA along the disease course would additionally promote the Th1 shift, thus closing a pathogenic vicious circle. In this scenario, our results point to DHA, EPA and stearic, a Th1-promoter NEFA associated with disease duration, as relevant mediators of this phenomenon, as they hallmarked the NEFA^low^ profile. In fact, the in vitro assays strongly support this idea. Hence, our findings shed some light on the involvement of NEFA as pivotal players for immune dysregulation on the basis of the Th1 response in RA. Therefore, strategies to counteract this NEFA imbalance would be advisable.

Other interesting findings of our study are the changes on NEFA levels upon TNFα-blockade. Our results identify that a balance between stearic and both EPA and DHA is related to a good clinical response in patients undergoing TNFα-blockade. Overall, our findings suggest that effective TNFα neutralization may counteract the disease duration-dependent NEFA impairment in RA. Moreover, changes in NEFA in good responders paralleled those of IFNγ. Therefore, our results suggest that a poor clinical outcome is related to a NEFA profile similar to NEFA^low^. Then, tailored FA supplementation to counteract decreasing NEFA over time could be advisable in these patients to increase the chance of achieving a good clinical response upon biological treatment, as recently suggested for triple-DMARD therapy [37]. Although largely explored, studies on FA supplementation in RA provided controversy results [38–40]. Overall, ω3-supplementation resulted in lower number of tender joints and pain scores [41], but no effect on disease activity or immunological parameters was reported. Thus, these effects can be attributed to an analgesic effect of ω3-derived products, whereas no effect on disease progression, the actual clinical goal, is achieved. Some studies revealed a lower NSAIDs consumption in the supplementation arm [39], thus supporting this idea. The results herein presented are particularly relevant for this issue. Since different NEFA profiles were observed, it is conceivable that differences in supplementation requirements exist in RA, thereby stressing the need for (i) an accurate stratification of patients according to their NEFA levels and (ii) a detailed design of FA formulas for supplementation. Actually, when similar supplementation interventions were performed, the effect was different among different conditions [42], thereby supporting the relevance of tailored strategies. In RA patients, stratification could be guided by clinical features as surrogate markers of NEFA impairment. Additionally, some concerns arise on the design of FA supplements, since not only dosages but also ratios among FA are crucial, following our results. Also, special attention needs to be paid when clinical trials are designed, since some included oleic acid as control, although it was found to be decreased in some diseases [43], including RA [38], where some beneficial effect was observed. Finally, chemical structure of FA for supplementation can have an impact on their clinical effects [44].

Our results suggest that EPA/DHA dose-dependently abrogate IFNγ release in resting and activated cells, thus supporting their use in supplementation schemes. There is limited evidence of the effect of FA supplementation on IFNγ in humans [45]. However, different FA dietary composition was found to modulate Th1-mediated bacterial clearance in mice [46]. Finally, ω3-supplementation would have a beneficial effect on cardiovascular (CV) outcomes [47], the most important comorbidity in RA. The link between NEFA^low^ profile and CCL2 may support this notion.

In conclusion, we report for the first time an altered serum NEFA profile in RA, which is associated with disease severity and enhanced Th1 CD4^+^ response. Equivalent results were obtained when patients undergoing TNFα-blockade were followed, impaired NEFA levels being related to a poor clinical outcome. Similarly, although some other NEFA may have an effect on cytokine production, we decided to primarily focus on stearic, EPA and DHA. Apart from being an objective data-driven approach, it led to the identification of individual compounds as responsible for the observed phenomena, with striking clues to unravel the disease mechanisms as well as to identify potential therapeutic targets. Therefore, our results shed some new light on the involvement of lipid species in RA pathogenesis and also provided a rationale for FA supplementation, stratification and formula-design in RA. However, a role for other NEFA or lipid species cannot be totally ruled out. Additional studies on lipid species on RA immunopathology are warranted.

## Supporting Information

S1 Fig Chromatograms of serum MTBE-extracted fractions. Total Ion Current chromatograms (TIC) from a representative healthy control (black line) and a RA patient (red line) are shown. Each peak represents a FA as follows: 1 (EPA), 2 (linolenic), 3 (γ-linolenic), 4 (palmitoleic), 5 (DHA), 6 (AA), 7 (linoleic), 8 (palmitic), 9 (oleic), 10 (heptadecanoic) and 11 (stearic). For peaks 1–4, a detailed Extracted Ion Chromatograms (EIC) corresponding to their specific m/z values are provided (right).
(TIF)

S2 Fig NEFA serum levels and disease duration. Analysis of the correlations between the disease duration and NEFA serum levels (stearic, palmitic, palmitoleic, AA, EPA and DHA) in RA patients. Correlations were assessed by Spearman rank’s correlation tests, and coefficient correlations and p-values are indicated for each analysis.
(TIF)

S3 Fig NEFA and IFNγ serum levels. Analysis of the correlations between serum levels of IFNγ and NEFA species (stearic, EPA and DHA) in RA patients. Correlations were assessed by Spearman rank’s correlation tests, and coefficient correlations and p-values are indicated for each analysis. IFNγ serum levels were log-transformed to facilitate visualization of these values.
(TIF)

S1 Table Individual and total NEFA serum levels in RA patients stratified according to disease stages. Serum levels of individual NEFA (μg/ml, measured by LC-MS/MS) and total NEFA (mM, measured by an enzymatic colorimetric assay) are summarized as median (interquartile range) and differences compared with HC were assessed by Mann Withney U test and indicated as *p<0.050, **p<0.010, ***p<0.001.
(DOCX)

S2 Table Individual and total NEFA serum levels in RA patients depending on their NEFA profile. Serum levels of individual NEFA (μg/ml, measured by LC-MS/MS) and total NEFA (mM, measured by an enzymatic colorimetric assay) are summarized as median (interquartile range) and differences were assessed by Mann Withney U test. Size effect was evaluated by Hedges’g statistic.
(DOCX)

S3 Table Individual and total NEFA serum levels in RA patients upon TNFα-blockade. Serum levels of individual NEFA (μg/ml, measured by LC-MS/MS) and total NEFA (mM, measured by an enzymatic colorimetric assay) are summarized as median (interquartile range) and differences were analyzed by paired T test.
(DOCX)

S4 Table Individual and total NEFA serum levels in RA patients upon TNFα-blockade stratified by their clinical response. Serum levels of individual NEFA (μg/ml, measured by LC-MS/MS) and total NEFA (mM, measured by an enzymatic colorimetric assay) are summarized as median (interquartile range) and differences were analyzed by paired T test.
(DOCX)
