# Supplementary figures and images for: Non-Esterified Fatty Acids Profiling in Rheumatoid Arthritis: Associations with Clinical Features and Th1 Response
Source: PLoS One. 2016 Aug 3;11(8):e0159573. doi: 10.1371/journal.pone.0159573 (PMC4972416; doi:10.1371/journal.pone.0159573)

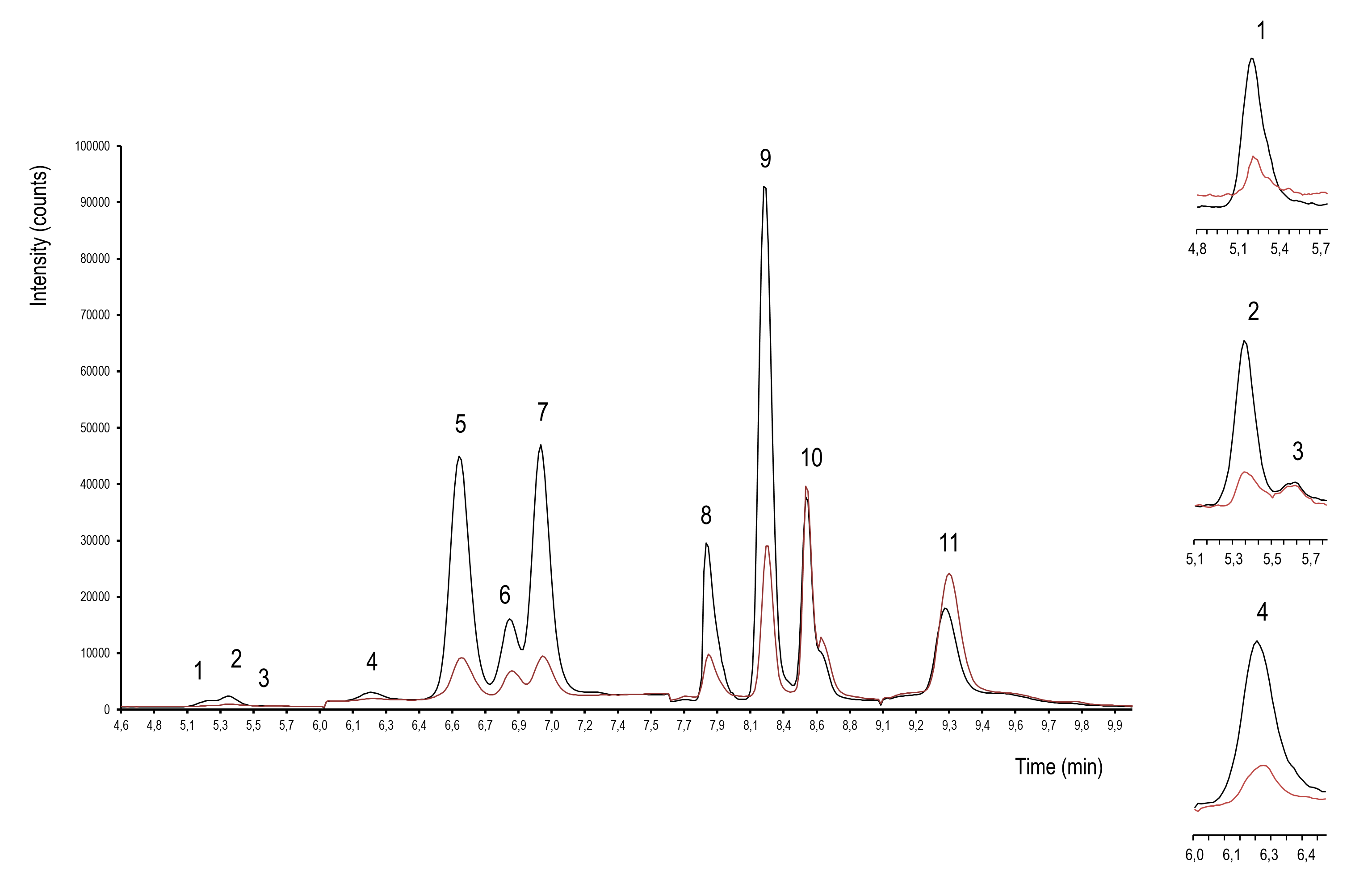

Supplement: S1 Fig — Total Ion Current chromatograms (TIC) from a representative healthy control (black line) and a RA patient (red line) are shown. Each peak represents a FA as follows: 1 (EPA), 2 (linolenic), 3 (γ-linolenic), 4 (palmitoleic), 5 (DHA), 6 (AA), 7 (linoleic), 8 (palmitic), 9 (oleic), 10 (heptadecanoic) and 11 (stearic). For peaks 1–4, a detailed Extracted Ion Chromatograms (EIC) corresponding to their specific m/z values are provided (right). (TIF) [file pone.0159573.s001.tif]

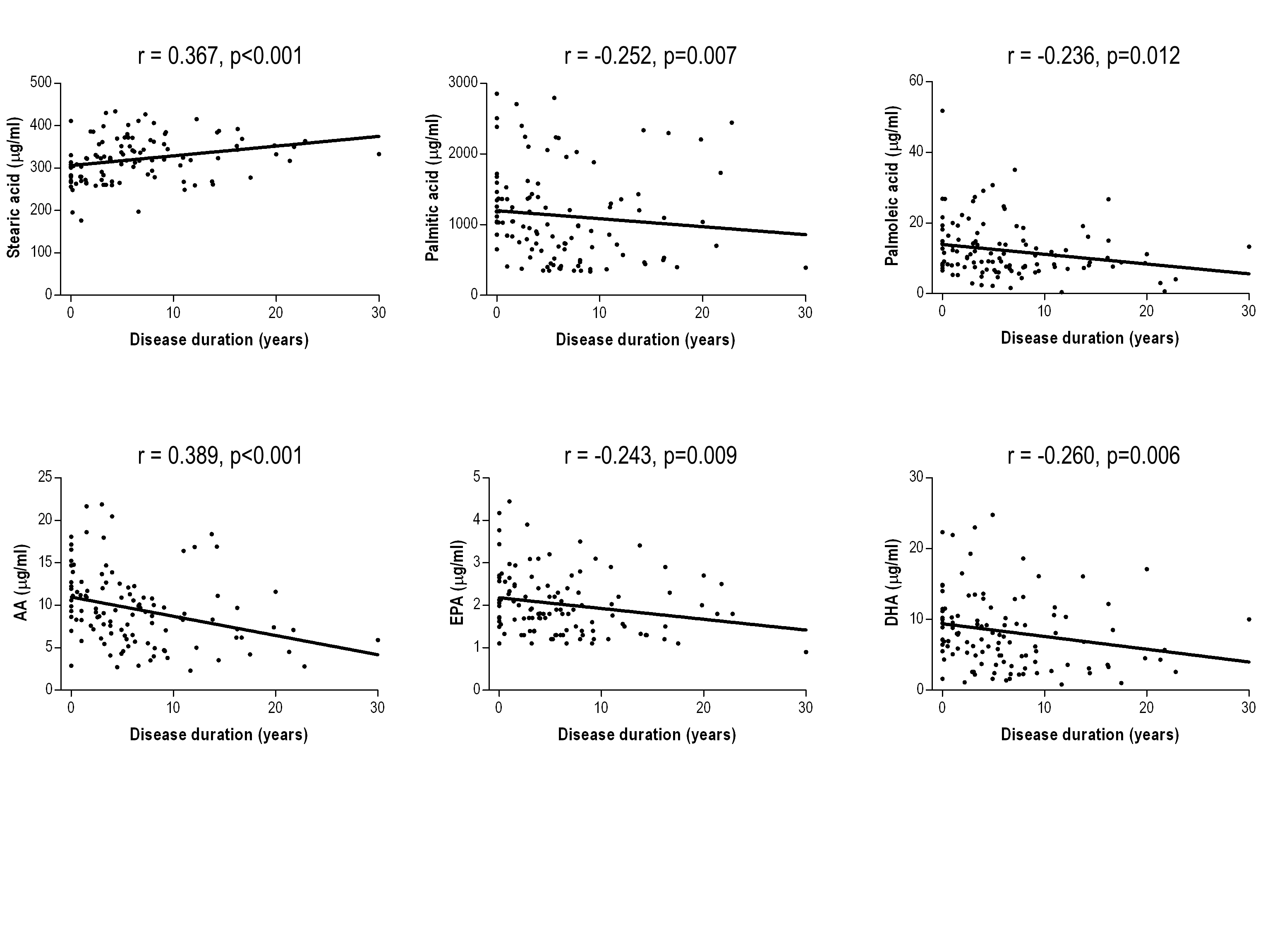

Supplement: S2 Fig — Analysis of the correlations between the disease duration and NEFA serum levels (stearic, palmitic, palmitoleic, AA, EPA and DHA) in RA patients. Correlations were assessed by Spearman rank’s correlation tests, and coefficient correlations and p-values are indicated for each analysis. (TIF) [file pone.0159573.s002.TIF]

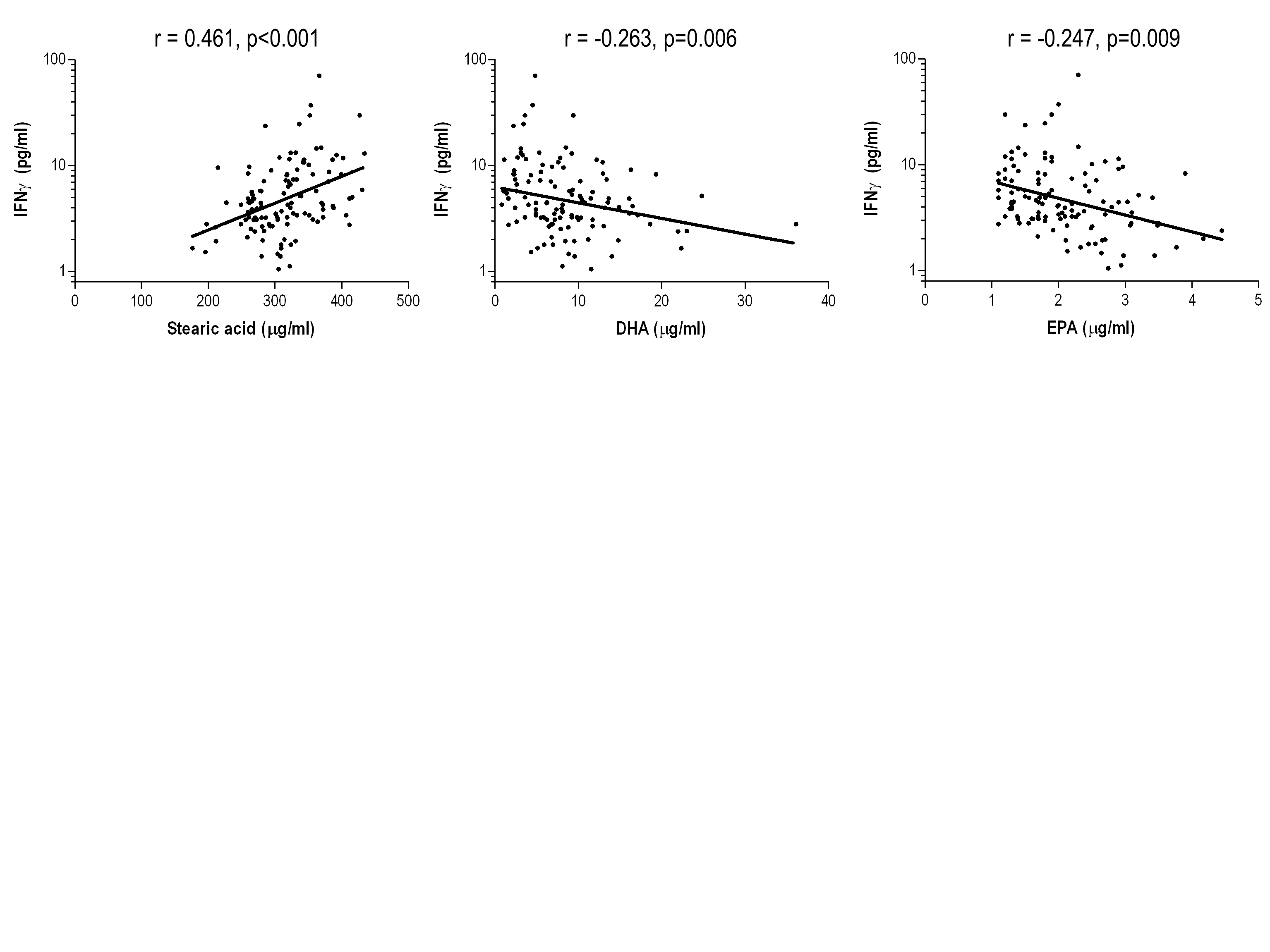

Supplement: S3 Fig — Analysis of the correlations between serum levels of IFNγ and NEFA species (stearic, EPA and DHA) in RA patients. Correlations were assessed by Spearman rank’s correlation tests, and coefficient correlations and p-values are indicated for each analysis. IFNγ serum levels were log-transformed to facilitate visualization of these values. (TIF) [file pone.0159573.s003.TIF]
