# Supplementary material for: Non-Esterified Fatty Acids Profiling in Rheumatoid Arthritis: Associations with Clinical Features and Th1 Response
Source: PLoS One. 2016 Aug 3;11(8):e0159573. doi: 10.1371/journal.pone.0159573 (PMC4972416; doi:10.1371/journal.pone.0159573)
Supplement: S1 Table — Serum levels of individual NEFA (μg/ml, measured by LC-MS/MS) and total NEFA (mM, measured by an enzymatic colorimetric assay) are summarized as median (interquartile range) and differences compared with HC were assessed by Mann Withney U test and indicated as *p<0.050, **p<0.010, ***p<0.001. (DOCX) [file pone.0159573.s004.docx]

**Supplementary Table 1:** Individual and total NEFA serum levels in RA patients stratified according to disease stages

| NEFA (μg/ml) | RA onset  (n=18) | Established RA  (n=106) |
| --- | --- | --- |
| Palmitic (16:0) | 1301.80 (648.36) | 910.00 (790.10) *** |
| Stearic (18:0) | 282.15 (38.78) | 321.87 (83.98) * |
| Palmitoleic (16:1w7) | 13.38 (11.61) | 9.89 (7.46) ** |
| Oleic (18:1w9) | 235.91 (246.54) | 193.40 (228.20) * |
| Linoleic (18:2w6) | 167.03 (206.20) | 143.10 (160.90) |
| γ-linoleic (18:3w6) | 1.43 (0.46) | 1.42 (0.48) |
| AA (20:4w6) | 12.06 (5.14) | 8.86 (5.01) ** |
| Linolenic (18:3w3) | 7.62 (3.00) * | 8.30 (4.98) |
| EPA (20:5w3) | 2.09 (1.03) | 1.90 (1.08) *** |
| DHA (22:6w3) | 9.74 (5.36) * | 7.36 (6.36) *** |
| Total NEFA (mM) | 0.41 (0.39) | 0.47 (0.38) |

Serum levels of individual NEFA (μg/ml, measured by LC-MS/MS) and total NEFA (mM, measured by an enzymatic colorimetric assay) are summarized as median (interquartile range) and differences compared with HC were assessed by Mann Withney U test and indicated as *p<0.050, **p<0.010, ***p<0.001.
