# Supplementary material for: Non-Esterified Fatty Acids Profiling in Rheumatoid Arthritis: Associations with Clinical Features and Th1 Response
Source: PLoS One. 2016 Aug 3;11(8):e0159573. doi: 10.1371/journal.pone.0159573 (PMC4972416; doi:10.1371/journal.pone.0159573)
Supplement: S2 Table — Serum levels of individual NEFA (μg/ml, measured by LC-MS/MS) and total NEFA (mM, measured by an enzymatic colorimetric assay) are summarized as median (interquartile range) and differences were assessed by Mann Withney U test. Size effect was evaluated by Hedges’g statistic. (DOCX) [file pone.0159573.s005.docx]

**Supplementary Table 2:** Individual and total NEFA serum levels in RA patients depending on their NEFA profile.

| NEFA (μg/ml) | NEFA^high^  (n=94) | NEFA^low^  (n=30) | *p-value* | *Hedges’ g* |
| --- | --- | --- | --- | --- |
| Palmitic (16:0) | 1194.87 (710.54) | 457.92 (210.67) | 2.07·10^-14^ | 1.44 |
| Stearic (18:0) | 303.83 (65.69) | 365.90 (66.00) | 6.18·10^-7^ | 1.18 |
| Palmitoleic (16:1w7) | 11.50 (10.22) | 8.15 (3.75) | 0.030 | 0.46 |
| Oleic (18:1w9) | 261.43 (262.6) | 129.05 (131.25) | 0.0003 | 0.73 |
| Linoleic (18:2w6) | 184.73 (186.10) | 100.45 (92.90) | 5.34·10^-5^ | 0.76 |
| γ-linoleic (18:3w6) | 1.49 (0.37) | 1.07 (0.56) | 0.001 | 0.43 |
| AA (20:4w6) | 9.91 (4.70) | 6.73 (4.13) | 2.52·10^-5^ | 0.60 |
| Linolenic (18:3w3) | 7.14 (3.35) | 11.55 (1.92) | 7.64·10^-11^ | 0.84 |
| EPA (20:5w3) | 2.20 (0.90) | 1.30 (0.30) | 2.03·10^-12^ | 1.45 |
| DHA (22:6w3) | 8.78 (5.97) | 3.60 (3.15) | 1.61·10^-9^ | 1.09 |
| Total NEFA (mM) | 0.48 (0.43) | 0.45 (0.25) | 0.691 |  |

Serum levels of individual NEFA (μg/ml, measured by LC-MS/MS) and total NEFA (mM, measured by an enzymatic colorimetric assay) are summarized as median (interquartile range) and differences were assessed by Mann Withney U test. Size effect was evaluated by Hedges’g statistic.
