# Supplementary material for: Non-Esterified Fatty Acids Profiling in Rheumatoid Arthritis: Associations with Clinical Features and Th1 Response
Source: PLoS One. 2016 Aug 3;11(8):e0159573. doi: 10.1371/journal.pone.0159573 (PMC4972416; doi:10.1371/journal.pone.0159573)
Supplement: S3 Table — Serum levels of individual NEFA (μg/ml, measured by LC-MS/MS) and total NEFA (mM, measured by an enzymatic colorimetric assay) are summarized as median (interquartile range) and differences were analyzed by paired T test. (DOCX) [file pone.0159573.s006.docx]

**Supplementary Table 3:** Individual and total NEFA serum levels in RA patients upon TNFα-blockade.

| NEFA (μg/ml) | t=0 | t=3 months | *p-value* |
| --- | --- | --- | --- |
| Palmitic (16:0) | 923.66 (192.70) | 833.57 (243.29) | 0.279 |
| Stearic (18:0) | 279.77 (93.35) | 280.01 (89.93) | 0.075 |
| Palmitoleic (16:1w7) | 14.72 (10.06) | 11.14 (10.36) | 0.552 |
| Oleic (18:1w9) | 204.63 (192.77) | 167.70 (263.49) | 0.552 |
| Linoleic (18:2w6) | 227.38 (155.58) | 130.67 (241.55) | 0.650 |
| γ-linoleic (18:3w6) | 1.56 (0.15) | 1.41 (0.30) | 0.182 |
| AA (20:4w6) | 9.85 (3.06) | 6.70 (3.61) | 0.002 |
| Linolenic (18:3w3) | 7.04 (2.64) | 6.68 (3.03) | 0.972 |
| EPA (20:5w3) | 2.64 (0.61) | 2.30 (0.33) | 0.009 |
| DHA (22:6w3) | 8.78 (3.71) | 5.40 (3.65) | 0.009 |
| Total NEFA (mM) | 0.50 (0.34) | 0.40 (0.47) | 0.214 |

Serum levels of individual NEFA (μg/ml, measured by LC-MS/MS) and total NEFA (mM, measured by an enzymatic colorimetric assay) are summarized as median (interquartile range) and differences were analyzed by paired T test.
