# Supplementary material for: Non-Esterified Fatty Acids Profiling in Rheumatoid Arthritis: Associations with Clinical Features and Th1 Response
Source: PLoS One. 2016 Aug 3;11(8):e0159573. doi: 10.1371/journal.pone.0159573 (PMC4972416; doi:10.1371/journal.pone.0159573)
Supplement: S4 Table — Serum levels of individual NEFA (μg/ml, measured by LC-MS/MS) and total NEFA (mM, measured by an enzymatic colorimetric assay) are summarized as median (interquartile range) and differences were analyzed by paired T test. (DOCX) [file pone.0159573.s007.docx]

**Supplementary Table 4:** Individual and total NEFA serum levels in RA patients upon TNFα-blockade stratified by their clinical response.

| NEFA (μg/ml) | t=0 | t=3 months | | *p-value* | |
| --- | --- | --- | --- | --- | --- |
| **Responders (n=5)** |  | |  |  | |
| Palmitic (16:0) | 1028.56 (207.16) | 927.47 (264.48) | | 0.225 | |
| Stearic (18:0) | 295.95 (55.92) | 273.28 (50.16) | | 0.043 | |
| Palmitoleic (16:1w7) | 15.28 (10.51) | 14.69 (15.55) | | 0.893 | |
| Oleic (18:1w9) | 290.72 (279.07) | 340.29 (340.22) | | 0.893 | |
| Linoleic (18:2w6) | 258.16 (174.43) | 195.95 (328.49) | | 0.500 | |
| γ-linoleic (18:3w6) | 1.63 (0.20) | 1.48 (0.33) | | 0.225 | |
| AA (20:4w6) | 11.28 (3.16) | 8.16 (3.52) | | 0.080 | |
| Linolenic (18:3w3) | 7.69 (4.00) | 7.98 (3.93) | | 0.500 | |
| EPA (20:5w3) | 2.94 (1.04) | 2.36 (0.97) | | 0.138 | |
| DHA (22:6w3) | 11.70 (10.51) | 8.83 (6.30) | | 0.225 | |
| Total NEFA (mM) | 0.50 (0.34) | 0.40 (0.47) | | 0.893 | |
| **Non responders (n=8)** |  |  | | |  |
| Palmitic (16:0) | 854.02 (142.82) | 781.55 (140.08) | | 0.484 | |
| Stearic (18:0) | 220.72 (86.7) | 247.01 (99.91) | | 0.327 | |
| Palmitoleic (16:1w7) | 13.45 (12.03) | 11.13 (8.36) | | 0.575 | |
| Oleic (18:1w9) | 185.99 (216.64) | 164.50 (229.57) | | 0.484 | |
| Linoleic (18:2w6) | 176.41 (118.78) | 126.66 (240.82) | | 1.000 | |
| γ-linoleic (18:3w6) | 1.55 (0.23) | 1.37 (0.37) | | 0.398 | |
| AA (20:4w6) | 9.59 (8.20) | 6.66 (4.43) | | 0.012 | |
| Linolenic (18:3w3) | 6.17 (2.64) | 6.65 (2.94) | | 0.575 | |
| EPA (20:5w3) | 2.57 (0.61) | 2.22 (0.28) | | 0.025 | |
| DHA (22:6w3) | 8.61 (2.89) | 4.98 (2.82) | | 0.025 | |
| Total NEFA (mM) | 0.50 (0.34) | 0.40 (0.47) | | 0.161 | |

Serum levels of individual NEFA (μg/ml, measured by LC-MS/MS) and total NEFA (mM, measured by an enzymatic colorimetric assay) are summarized as median (interquartile range) and differences were analyzed by paired T test.
